# New biotechnology enhances the application of cisgenesis in plant breeding

**DOI:** 10.3389/fpls.2014.00389

**Published:** 2014-08-11

**Authors:** Hongwei Hou, Neslihan Atlihan, Zhen-Xiang Lu

**Affiliations:** ^1^Institute of Hydrobiology, Chinese Academy of SciencesWuhan, China; ^2^Lethbridge Research Centre, Agriculture and Agri-Food CanadaLethbridge, AB, Canada; ^3^Central Research Institute of Food and Feed ControlBursa, Turkey

**Keywords:** cisgenesis, crop breeding, genetically modified organism, safety evaluation, plant biotechnology

## Abstract

Cisgenesis is genetic modification to transfer beneficial alleles from crossable species into a recipient plant. The donor genes transferred by cisgenesis are the same as those used in traditional breeding. It can avoid linkage drag, enhance the use of existing gene alleles. This approach combines traditional breeding techniques with modern biotechnology and dramatically speeds up the breeding process. This allows plant genomes to be modified while remaining plants within the gene pool. Therefore, cisgenic plants should not be assessed as transgenics for environmental impacts.

## INTRODUCTION

Increasing world population and food demands require world agricultural production be increased by 50% by 2030 (The Royal Society, 2009). In the meantime, climate change and shrinking environmental resources are limiting agricultural production over the world (Lobell et al., 2008). These challenges bring an urgent need to enhance crop productivity. To breed crops with increased yield and resistance to environment stresses, a pivotal consideration is how to effectively utilize genetic diversity.

Genetic crossing, selection of natural or artificial mutations, and transgenics, are the main techniques for plant breeding. Traditional plant breeding uses crossing, mutagenesis and somatic hybridization for genome modification to improve crop traits. It introduces new beneficial alleles from crossable species. Due to crossing barriers and linkage drag, however, traditional plant breeding is time-consuming and requires several generations of breeding and selection. Transgenic breeding uses molecular cloning techniques to identify cloned or synthesized genes of interest and directly transforms the recipient genome. This process manipulates plant genomes through insertion of gene(s) from another species. An organism that is generated this way is considered to be a genetically modified organism (GMO). Transgenic plants have been created with better tolerance against natural stresses, and to produce biofuels, vaccines, and antibodies (Ahmad et al., 2012). Many plant species, including major crops such as rice, soybean, maize, cotton, canola, potato, cassava, squash, papaya, groundnut, oilseeds, and numerous vegetables and fruits have foreign genes inserted in their genomes (Mendoza et al., 2008; Chakraborty et al., 2010; Hutchison et al., 2010; Llorente et al., 2010; Motoyama et al., 2010; Asif et al., 2011). However, transgenic crops have brought considerable concerns about their safety and impact on health and the environment. This is because exogenous genes from other species such as bacteria, or synthesized DNA such as DNA borders are transferred to plant genomes, and the sites of insertion are random, which may have unpredictable side effects.

Recent progress in plant genome sequencing facilitates the isolation of plant genes from crossable species. These genes are called cisgenes. Increasing numbers of these cisgenes have been isolated and new transformation protocols have been developed, which do not leave marker genes behind. This allows plant genomes to be modified while remaining plants within the gene pool. This new breeding approach was first called cisgenesis by Schouten et al. (2006), who defined a cisgenic plant as “a crop plant that has been genetically modified with one or more genes isolated from a crossable donor plant.” In the scientific opinion of the [European Food Safety Authority (EFSA), 2012], cisgenesis is described as specific alleles/genes in the breeder’s gene pool are introduced into new varieties without the accompanying linkage drag (co-transfer of DNA sequences that are linked to the gene of interest) which occurs in conventional breeding. In contrast, a transgenic plant receives gene(s) from a non-plant organism, or from a donor plant that is sexually incompatible with the recipient plant. For example, the introduction of the *R1* gene, the first gene for resistance to late-blight, from wild type potato (*Solanum demissum)* to cultivated potato (*S. tuberosum)* is cisgenesis. However, the transfer of the *Bt* gene from the bacteria *Bacillus thuringiensis* to cotton genome to produce pest resistant cotton is transgenesis.

Cisgenic plants are more likely to be acceptable to the public than transgenic plants. According to a survey in the USA, 55–77% of people who participated would eat a cisgenic or intragenic vegetable (depending on number of genes and source of the gene) whereas 17–25% of them would eat the vegetable if it contained a gene from a microorganism or an animal. In another survey in Mississippi, 81% would eat a cisgenic or intragenic vegetable, while only 14–23% would eat a transgenic vegetable containing non plant sources (Viswanath and Strauss, 2010). In the EU, cisgenic plants are expected to be more acceptable for consumers (Herzog, 2012).

## CISGENESIS IS DRAMATICALLY IMPROVING EFFICIENCY IN USE OF SUPERIOR ALLELES

In traditional breeding, obtaining desired traits is painstakingly slow and one challenge is the low affinity between wild and cultivated varieties. Techniques such as embryo rescues, cross bridges and somatic cell hybridization help overcome this obstacle. An example of their use is in breeding late-blight resistant potato. By using traditional breeding, Hermsen and Ramanna (1973) overcame numerous obstacles to establish double bridge hybrids of *S. bulbocastanum* and cultivars of *S. tuberosum*. They used wild varieties of *S. acaule* and *S. phureja* as bridge parents to introduce the late-blight resistance trait from *S. bulbocastanum* to cultivars of *S. tuberosum,* resulting in so-called ABPT material, which is used widely for breeding late-blight resistant potato varieties. The first late-blight resistant potato variety “Biogold” was developed and released 30 years later. In contrast to such long term traditional breeding, van der Vossen et al. (2003, 2005) applied the cisgenesis breeding approach and successfully cloned and introduced three late-blight resistance genes from *S. bulbocastanum to* cultivated potato within only a few years.

Another obstacle in traditional breeding is linkage drag. This problem can only be solved by many generations of backcrosses and screening for recombination, which is uncoupling the wanted trait from those traits that are unwanted (Jacobsen and Schouten, 2007). Such a process is time-consuming, especially for crops with a long generation period. The problem is even more serious if several genes from different wild sources have to be accumulated, e.g., for durable resistance. This could be prevented if only the gene(s) of interest was added, leaving the undesired genes in the wild germplasm behind. Using isolated cisgenes coding only for wanted traits could drastically overcome these problems, because cisgenesis allows genes containing their native introns, promoters, and terminators to be introduced into cultivated varieties. An example is breeding apples for scab (*Venturia inaequalis*) resistance. Introgression of the apple scab resistance gene *Vf* from *Malus floribunda* 821 into marketable high quality apple cultivars started in the 1950s (Hough et al., 1953), and breeding continued for more than 50 years. This slow tempo is mainly caused by the long juvenile period of apple trees and the phenomenon that not only the allele of interest is inherited by the progeny, but also hundreds of unwanted alleles. For durable resistance, different resistance genes must be accumulated, but this may take a few more decades. The process would be much faster to achieve by cisgenesis, which combines the knowledge of native alleles with marker-free technologies and allows the introduction of resistance-gene alleles without associated selection genes.

There are a number of examples of the successful use of cisgenesis in developing improved crops. In a project named Durable Resistance against *Phytophthora* (DuRP), a cisgenic strategy was used to develop potatoes resistant to late-blight. Haverkort et al. (2009) tried several ways to clone genes from susceptible and resistant plants of selected wild species. The R genes or a cassette of several R genes were transferred to potato leaf cells via *Agrobacterium tumefaciens*. A marker was used only for the fast determination of the best R gene combination. After that step, the desired potatoes with these genes were modified in a marker-free way. The field trials of potato crops produced during this project are ongoing in Belgium, Ireland, and the Netherlands (Holme et al., 2013).

Cisgenesis has also been successfully applied in cereal crops and trees. In barley, a marker-free cisgenic variety has been developed, with an extra phytase gene introduced to enhance phosphate bioavailability by degrading phytic acid enzymatically (Holme et al., 2012). Using the pClean dual binary vector system with hygromycin resistance for selection, two T1 lines were identified containing a single purple acid phosphatase (PAP) insert (both hygromycin and kanamycin resistance genes had been removed). Field trials are being carried out in Denmark, with a 2012–2016 release period (Holme et al., 2013). In durum wheat, Gadaleta et al. (2008) conducted biolistic transformation to enhance bread making properties. Wheat D genome genes encoding the 1D × 5 and 1 Dy10 glutenin subunits with their own native endosperm promotors and terminators were cloned and transferred. As a positive selectable marker, *Escherichia coli* phosphomannose isomerase (pmi) was removed from minimal gene cassettes under genetic segregation and positive selection. In poplar trees, the effects on plant growth from insertion of five cisgenes that encode proteins for gibberellin metabolism or signaling have been studied (Han et al., 2011). All parts of cisgenes, including their promoter and terminator, were transferred into the *Populus trichocarpa* genome. Three of the cisgenic modifications had significant effects on either plant growth rate, morphology or wood properties.

Thus cisgenic insertion of additional copies of native genes may provide a new approach to modify plant genomes, expand genetic variance in plant architecture available to breeders and accelerate the transfer of alleles between species which are difficult to cross.

## NEW BIOTECHNOLOGY IS MAKING CISGENESIS INCREASINGLY FEASIBLE IN USE OF GENE RESOURCES

The bottleneck in modern crop breeding is the limited genetic variability in any one crop species, since the plant breeding process has selected only superior genotypes in which the genetic variation is reduced (Simmonds, 1993; Tanksley and McCouch, 1997). Such limitation of crop genetic variations is a major concern because it may result in widespread losses in crop yield and quality if new pathogen populations or unusual abiotic stresses occur. Incorporation of new alleles from wild germplasm that confer pathogen resistance, can alleviate this genetic vulnerability (Maunder, 1992; Cox and Wood, 1999).

Plant genome sequencing facilitates the isolation of cisgenes from crossable crops or wild species. Cost-effective high-throughput sequencing techniques have been invented that allow a whole plant genome to be sequenced in a few days rather than a few years as in the past. From just a few model species originally, now whole genomes of a wide variety of organisms can be sequenced. Currently, genomes of more than 180 organisms have been sequenced since 1995. This provides more varieties of gene resources for manipulation, which facilitates the isolation of cisgenes from crossable crops or wild species. In the meantime, efficient gene isolation methods, such as map-based cloning and allele mining, open new avenues in plant breeding by using cloned indigenous genes. Furthermore, advances in plant molecular biology have greatly facilitated the isolation of plant genes associated with economically important traits (Pereira, 2000), which provides alternative approaches to those that rely on genetic variation that has evolved in unrelated species.

## NEW BIOTECHNOLOGY IS MAKING CISGENESIS INCREASINGLY FEASIBLE FOR PRECISELY OBTAINING NEW AGRICULTURAL TRAITS

To avoid the introduction of exogenous genes, new transformation protocols, without bacterial selection markers, have been developed (McKnight et al., 1987; de Vetten et al., 2003; Schaart et al., 2004). To circumvent the need for using bacterial T-DNAs, the isolated genes are inserted into species-specific P-DNAs (de Vetten et al., 2003; Rommens, 2004).

Application of new methods, such as promoter trapping and RNA fingerprinting, has resulted in the isolation of native regulatory elements that can now be exploited for the precise expression of the desired traits (Meissner et al., 2000; Trindade et al., 2003).

Gene editing techniques, such as the use of zinc finger nucleases (ZFNs) and transcription activator-like effectors nucleases (TALEN), allow site-directed modifications in the genes of interest. ZFNs are hybrid enzymes composed of a naturally occurring FokI restriction endonuclease and zinc finger DNA binding domains (Curtin et al., 2012). Zinc finger proteins recognize and bind to target sequences and FokI nuclease catalyze cleavage of DNA base pairs downstream of the recognition site. Each zinc finger protein recognizes three particular base pairs in target DNA (de Pater et al., 2009). In *Arabidopsis*, de Pater et al. (2009) generated ZFNs with six zinc fingers which can recognize 18 base pairs in target DNA to introduce mutations. ZFNs has also been used to induce mutations in tobacco (Townsend et al., 2009) and maize (Shukla et al., 2009). TALEN are proteins produced by the plant pathogenic bacterium *Xanthomonas* and bind to specific DNA sequences (Curtin et al., 2012). Together with endonuclease FokI, TALEN has been used to introduce mutations in *Arabidopsis*, tobacco protoplasts and leaves, rice and *Brachypodium* (Curtin et al., 2012; Shan et al., 2013; Zhang et al., 2013). There are several open engineering platforms available for construction of ZFNs and TALENs (Townsend et al., 2009; Curtin et al., 2012; Zhang et al., 2013).

Recently, the clustered regulatory interspaced short palindromic repeats (CRISPR) associated nuclease (Cas) system has been reported to be an efficient and highly specific method for gene editing (Feng et al., 2013; Upadhyay et al., 2013). Cas is a protein catalyzing DNA cleavage. CRISPR consists of variable short spacer sequences separated by short repeat sequences and these CRISPR arrays are transcribed into non-coding RNAs (Jinek et al., 2013). CRISPR-Cas mediated genome editing has been reported in wheat (*Triticum aestivum*) and *Nicotiana benthamiana* (Upadhyay et al., 2013), as the mutations were induced in *inositol oxygenase* (*inox*) and *phytoene desaturase (pds)* genes in wheat and *pds* gene in *N. benthamiana*, respectively. This system has also been used to introduce mutations in the OsMPK5 gene which encodes a stress-responsive protein kinase in the rice genome (Xie and Yang, 2013) and the mutations of several genes in *Arabidopsis* and rice (Feng et al., 2013).

Several methods have been developed to remove selectable marker genes from the genomes of transgenic plants. One approach exploits an inducible recombination system to excise a marker gene positioned between recombination sites (Zuo et al., 2001) and another strategy relies on the segregation of independently integrated T-DNAs (Komari et al., 1996). New and efficient *Agrobacterium*-based methods that utilize a plant-derived transfer DNA and a novel transient selection system to insert only native DNA into potato plants have been invented (Rommens et al., 2004). However, the majority of these methods for production of cisgenic crops have been patented, therefore scientists need either to use these patents or design new methods to eliminate the undesired DNA sequences from host genomes (Holme et al., 2013).

## CISGENIC PLANTS SHOULD BE DISTINGUISHED FROM TRANSGENIC PLANTS

The release of genetically modified (GM) plants is currently regulated to prevent any negative effects on the environment or human health. These regulations are based on transgenic organisms and do not distinguish transgenic plants from cisgenic plants. This means that the GM-regulations for transgenes (genes from the non-crossable species), are also applied for cisgenes (genes from crossable species). However, cisgenesis is more similar to traditional plant breeding than is transgenesis. There is a great necessity to distinguish cisgenesis from transgenesis.

Although both transgenesis and cisgenesis use the same genetic modification techniques to introduce gene(s) into a plant, cisgenesis introduce only genes of interest from the plant itself or from a crossable species, and these genes could also be transferred by traditional breeding techniques. Therefore, cisgenesis is not any different from traditional breeding or that which occurs in nature. There is no environmental risk evoked and release of cisgenic plants into the environment is as safe as that of traditionally bred plants.

If the current international GMO regulations continue to fail in distinguishing cisgenic from transgenic plants, the use of cisgenesis could be seriously hindered. Only Canada now has a product-based regulation system rather than a process-based one and this has made it legally possible to control cisgenic plants less strictly than transgenic plants. Any restrictions on cisgenesis could block or delay further research and application of improved crop varieties, especially at a time when increasing number of genes from crops and their crossable wild relatives are being isolated and are becoming amenable to cisgenesis. In Australia, cisgenic plants are treated differently under GMO regulations, as stated in Gene Technology Regulations that “a mutant organism in which the mutational event did not involve the introduction of any foreign nucleic acid” is not specified as GMO (Russell and Sparrow, 2008).

European Food Safety Authority has released a scientific assessment of the safety assessment of plants developed through cisgenesis and intragenesis. According to this report concerning the source of introduced genes, cisgenesis has similar hazards as does traditional breeding. However, the transformation techniques used in cisgenesis and transgenesis are the same, so they have similar risk linked to transfer technology. This report recommended to using the same risk assessment guides as used in transgenic plants to evaluate the cisgenic plants, but the required information might be less than that needed for transgenic plants [European Food Safety Authority (EFSA), 2012].

## CONCLUDING REMARKS AND PERSPECTIVES

Traditional breeding provides us excellent plants with many genes working together in a concerted manner. Plant breeders may have a limited knowledge of the underlying genetic networks, but they are still able to develop superior crop cultivars. Because of the complexity of plant functions, traditional breeding has been widely used and will remain crucially important for agricultural production. Cisgenesis is the transfer of gene(s) from the recipient plant itself, or from a donor plant that is sexually compatible with the recipient plant. Knowledge of traditional breeding remains critical for selection of cisgenic plants in breeding by cisgenesis. New biotechnology is making cisgenesis increasingly feasible in use of gene resources and precisely obtaining new agricultural traits without insertion of foreign genes or gene fragments.

## Conflict of Interest Statement

The authors declare that the research was conducted in the absence of any commercial or financial relationships that could be construed as a potential conflict of interest.
